# FXR deficiency in hepatocytes disrupts the bile acid homeostasis and inhibits autophagy to promote liver injury in *Schistosoma japonicum*-infected mice

**DOI:** 10.1371/journal.pntd.0010651

**Published:** 2022-08-05

**Authors:** Beibei Zhang, Jing Li, Xianlong Zong, Jianling Wang, Lianlian Xin, Haiyao Song, Wenxue Zhang, Stephane Koda, Hui Hua, Bo Zhang, Qian Yu, Kui-Yang Zheng, Chao Yan

**Affiliations:** 1 Jiangsu Key Laboratory of Immunity and Metabolism, Department of Pathogenic Biology and Immunology, Laboratory of Infection and Immunity, Xuzhou Medical University, Xuzhou, People’s Republic of China; 2 National Demonstration Center for Experimental Basic Medical Science Education, Xuzhou Medical University, Xuzhou, People’s Republic of China; 3 School of Stomatology, Xuzhou Medical University, Xuzhou, People’s Republic of China; Instituto de Salud Carlos III, SPAIN

## Abstract

**Background:**

Schistosomiasis, with 250 million people affected, is characterized by its serious hepatic inflammatory response and fibrosis formation, which could lead to dangerous complications, such as portal hypertension, splenomegaly and even ascites. But until now, the pathogenesis of schistosomiasis remains largely unknown. Farnesoid X Receptor (FXR), a bile acid-activated nuclear transcription factor mainly expresses in hepatocytes in the liver, can regulate liver diseases by controlling bile acid metabolism.

**Methodology/Principal findings:**

In this study, we found that the expression of FXR was decreased in the liver of infected mice as shown by western blot and RT-qPCR assays. Furthermore, hepatocyte-specific FXR-deficient mice (*FXR^flox/flox^Alb^Cre^*, FXR-HKO) were generated and infected with ~16 cercariae of *S*. *japonicum* for five weeks. We found that FXR deficiency in hepatocytes promoted the progression of liver injury, aggravated weight loss and death caused by infection, and promoted inflammatory cytokines production, such as IL-6, IL-1β, TNF-α, IL-4, IL-10, and IL-13. Surprisingly, hepatic granulomas and fibrosis were not affected. In addition, using UPLC-MS/MS spectrometry, it was found that *S*. *japonicum* infection resulted in elevated bile acids in the liver of mice, which was more obvious in FXR-deficient mice. Meanwhile, autophagy was induced in littermate control mice due to the infection, but it was significantly decreased in FXR-HKO mice.

**Conclusions/Significance:**

All these findings suggest that FXR deficiency in hepatocytes disrupts bile acid homeostasis and inhibits autophagy, which may aggravate the damages of hepatocytes caused by *S*. *japonicum* infection. It highlights that FXR in hepatocytes plays a regulatory role in the progression of schistosomiasis.

## Author summary

The liver, a critical metabolic organ, consists of approximately 80% parenchymal hepatocytes. Hepatic schistosomiasis results in inflammatory granulomas response and fibrosis formation that inevitably affects hepatocytes. However, whether and how hepatocytes involved in the progression of liver injury caused by *S*. *japonicum* is not clear. Here, we found that, the level of FXR, a key regulator of bile acid metabolism in hepatocytes, was clearly decreased in the liver of mice with this worm infection. Further, we found that FXR specifically deficient in hepatocytes increased bile acids toxicity and inhibited hepatocellular autophagy induced by worm infection, which therefore accelerated the progression of schistosomiasis by promoting hepatocyte injuries but not the formation of egg granulomas and hepatic fibrosis. Our work provides a regulatory loop of FXR/bile acids-autophagy in schistosomiasis, which suggests a role of hepatic FXR in the protection from hepatic damages caused by infection with *S*. *japonicum*.

## Introduction

Schistosomiasis infecting 250 million people is a debilitating neglected tropic disease (NTDs) caused by infection with *Schistosoma spp* [1]. It can lead to dangerous complications, such as occlusion of the portal veins, portal hypertension, splenomegaly and even ascites. The *Schistosoma japonicum* (*S*. *japonicum*) is the main species distributed in East Asia such as China and Philippines [2]. Hepatic schistosomiasis caused by *S*. *japonicum* infection is characterized by early inflammatory granulomas response and late fibrosis formation in the periportal spaces. It is well known that interleukin 4 (IL-4) and IL-13-mediated type II immune response leads to the development of granulomas around eggs trapped [3]. Then the quiescent hepatic stellate cells (HSCs) are activated and recruited to the edge of granulomas, which produce extracellular matrix and fibrillar collagens to result in hepatic fibrosis [4]. In addition, the activated HSCs and fibrillar collagens are close to the gap in the endothelial cells, and impede solutes from the blood to the hepatocytes. Thus, it is inevitable to result in ischemic necrosis of parenchymal hepatocytes [5]. Hepatocytes consist of more than 80% of the liver cells, which is a major functional portion in the liver microenvironment under diseased conditions. Wang and his colleagues reported that hepatocytes promoted liver fibrosis triggered by schistosomiasis in a Wnt signaling-dependent manner [6]. This suggests that the potential role of parenchymal hepatocytes in the pathology of schistosomiasis could not be ignored.

As a critical metabolic organ, liver bile acid metabolism is known for its “classical” roles in absorption of dietary nutrition and cholesterol homeostasis. Recently, the functional roles of bile acids have been largely revealed. Bile acids can work as signaling molecules that regulate the balance of glucose, lipid and energy metabolism, control the detoxification reactions, and modulate the immune response via activating nuclear receptors and membrane G protein-coupled bile acid receptors [7, 8, 9, 10]. The nuclear receptor subfamily 1 group H member 4 (NR1H4), also called Farnesoid X Receptor (FXR), is a bile acid-activated nuclear transcription factor that belongs to nuclear factors. FXR is widely expressed in hepatocytes, cholangiocytes, and immune cells including Kupffer cells, T helper cells, NKT cells and dendritic cells [8]. FXR keeps inactivated under physiological conditions, however, knocking out of FXR contributes to the development of nonalcoholic fatty liver disease, nonalcoholic steatohepatitis, hepatic fibrosis, and even hepatocellular carcinoma [11, 12]. It has been reported that FXR negatively regulates the NLRP3 inflammasome to control cholestasis-associated sepsis [13]. All this highlights the importance of FXR in the healthy and diseased liver. The inflammatory cells infiltrations, fibrosis formation, and injured hepatocytes are found due to infection with *S*. *japonicum*. However, whether bile acid/FXR signaling is involved in this process or not remains unknown.

Autophagy is a highly conserved lysosomal degradation pathway that is crucial to organelle turnover, clearance of abnormal protein aggregates and invaded microorganisms. It keeps at a basal level to maintain physiological homeostasis in all cells, but is up-regulated rapidly in response to stress. Aberrant autophagy can promote the progression of multiple liver diseases such as viral hepatitis, nonalcoholic fatty liver disease, and hepatocellular carcinoma [14]. It was reported that autophagy was suppressed in cholestasis by overloaded bile acids in hepatocytes, which was regulated by bile acid receptor FXR [15, 16]. Inhibition of FXR activity impairs the autophagy flux and potentiates bile acids cytotoxicity, which indicates that FXR can modulate autophagy and protects hepatocytes in cholestatic livers [17]. However, the mechanism by which FXR regulates autophagy to prevent hepatocytes injuries caused by infection with *S*. *japonicum* is still obscure. In the present study, we prepared a hepatocyte-specific FXR KO mouse using the Cre-flox system (*Alb*^Cre^ crossed with *FXR^flox/flox^*), followed by the infection of cercariae *S*. *japonicum*, we found that hepatocyte-specific FXR KO promoted weight loss and death of mice with schistosomiasis. Furthermore, FXR deficiency aggravated the hepatocytes injury, which may be associated with the increased bile acids toxicity and inhibited hepatocellular autophagy. However, it didn’t affect pathologies of eggs granulomas and hepatic fibrosis. This work highlights that FXR in hepatocytes plays a role in the hepatic damages caused by *S*. *japonicum* infection.

## Methods

### Ethics statement

All animal experiments were performed with the approval of the Guide for the Care and Use of Laboratory Animals of the National Institutes of Health. The experiments were carried out in strict accordance with the guidelines of the Committee for Animal Research of Xuzhou Medical University (201801w003).

### Hepatocyte-specific FXR KO mice and infection

Hepatocyte-specific FXR KO mice (FXR-HKO) were generated by crossing the floxed NRIH4 (also known as *Fxr* exon 9) (C57BL/6-*Nr1h4^em1(flox)Smoc^*; Jackson Laboratories) C57BL/6J mice with mice containing the Albumin Cre promoter *(Alb-Cr*e). Mice carrying loxP-flanked NRIH4 (Fxr*^flox/flox^* alleles were used as wild-type (WT) mice. Mice were bred in a specific pathogen-free facility with light and temperature-controlled environment at the animal center of Xuzhou Medical University. The phenotypes of mice used for *S*. *japonicum* infection were genotyped by PCR and confirmed using western blot at the protein level. The experiments were carried out in strict accordance with guidelines of the Committee for Animal Research of Xuzhou Medical University (201801w003).

For establishing *S*. *japonicum* infected mice, *Oncomelania hupehensis* snails with cercariae infection were obtained from the National Institute of Parasitic Diseases, Chinese Center for Disease Control and Prevention in Shanghai. The cercariae were released from the snail by putting the snail into water under light. For establishing infected mice model with different time-courses, male mice were randomly divided into six groups (n = 5 for each group), including Normal group, 4 weeks post-infection group (4 wpi), 6 wpi, 8 wpi, 10 wpi, and 14 wpi. For establishing the infected FXR-HKO mice model, *Fxr^flox/flox^* (for littermate control, WT) and FXR-HKO mice were randomly divided into two groups, respectively (n = 7 for each group). In the infected group, each WT and FXR-HKO mice were infected with ~16 cercariae. In detail, cercariae with high vitality were collected on a coverslip and counted under the anatomical microscope. Then, the coverslide was put on the bare abdomen of the mouse for at least 15 minutes. After five-week of post-infection (5 wpi), mice were kept in deep anaesthesia with 2% pentobarbital and sacrificed for collecting samples.

### Histology and immunofluorescence

Liver tissues were collected and fixed in 4% paraformaldehyde overnight. Then, the paraffin-embedded tissues from seven mice of each group were sectioned, dewaxed and hydrated. HE staining and Masson staining were conducted for eggs granulomas and fibrosis analysis, respectively. Pictures were obtained by an Olympus IX51 microscope. The percentages of eggs granulomas and fibrosis were analyzed by Image-Pro Plus 6.0 software.

Autophagy was determined with immunofluorescence using LC3 antibody (3868, Cell Signaling Technology, Boston, United States). In detail, sections with 4 μm thickness were dewaxed and gradiently hydrated. After washing with PBS three times, 3% hydrogen peroxide was used to inactive the endogenous peroxidase for 10 minutes. And 5% bovine serum albumin was used for blocking sections for 1 hour. The primary LC3 antibody (1:200) was added to the sections and incubated at 4°C overnight. PBS was used as a negative control. Then, the corresponding secondary antibody (SA00013-4, Proteintech, Chicago, United States) was applied for 1 hour incubation. After washing, sections were covered with an anti-fade mounting medium containing DAPI. Images were captured by an Olympus IX51 microscope. Cells with LC3 dots were numbered.

### Egg counts

The egg count was performed as previously reported [18]. In brief, each liver sample (200 mg) was digested in 1 ml of 4% KOH overnight at 37°C in a shaker. Eggs were counted under an optical microscope by putting 10 μl of suspension on to a slide and repeated five times. The number of eggs per gram was calculated.

### Biochemical analysis

Serum was isolated by centrifuging the blood at 3500 rpm for 15 minutes. Alanine transaminase (ALT), aspartate aminotransferase (AST), and total bile acid (TBA) were detected by ROCHE Cobas 8000 automatic biochemical analyzer in the laboratory of Affiliated Hospital of Xuzhou Medical University.

### Ultra performance liquid chromatography/tandem mass spectrometry

Total bile acid was extracted by homogenizing 200 mg of liver sample in 400 μl of distilled deionized water. The supernatant was fixed with an equal volume of ice acetonitrile, and vortexed for 25 min at room temperature. Then, the supernatant was collected after centrifuging at 12000 g for 15 min. Extract (10 μl) was analyzed using a UPLC-MS/MS spectrometry (Xevo TQ-S micro, Waters, Massachusetts, United States). UPLC was achieved using solvent A (500 ml of ultrapure water, and 0.385 g of ammonium acetate) and solvent B (500 μl of formic acid, and 500 ml of acetonitrile). Separation was performed using a BEH C18 column maintained at 45°C. The spectrometry was made in electrospray negative mode with a capillary voltage of 2500V at 450°C. The flow rate of dissolvent gas was 800 L/h, reverse cone-hole gas: 50 L/h, and collision gas: 0.15 ml/min. MassLynx V4.1 software was applied to monitor the mass fragmentation and integrated detected peak areas relative to the internal standards.

### Enzyme-linked immunosorbent assay

ELISA was employed to detect cytokines of interleukin (IL)-6 (88–7064, Invitrogen, California, United States), IL-1β (88–7013, Invitrogen, California, United States), IL-4 (88–7044, Invitrogen, California, United States), IL-10 (88–7105, Invitrogen, California, United States), IL-13 (88–7137, Invitrogen, California, United States), and tumor necrosis factor α (TNF-α) (88–7324, Invitrogen, California, United States). Briefly, 200 mg of liver tissue was homogenated with 1 ml of RIPA (P0013B, Beyotime, Shanghai, China) and 1% protease inhibitor (P1082, Beyotime, Shanghai, China). The supernatant was collected for the determination of total protein concentration. The procedure of ELISA was followed the manufacturer’s instructions. Concentrations of these cytokines were calculated according to the standard curves and normalized to the total protein concentration.

### RNA isolation and RT-qPCR

RNA was isolated from liver tissues with TRIzol reagent (DP424, TIANGEN, Beijing, China). Nanodrop 2000 spectrophotometer was used to determine the quality and quantity of RNA. Then, 3 μg RNA was transcribed into cDNA followed by first strand synthesis and reverse transcription with a first-strand cDNA Synthesis Kit (11141ES60, YEASEN, Shanghai, China). RT-qPCR assays were performed as the following reaction procedure: 95°C for 5 min; 35 cycles of 95°C for 10 s, 60°C for 10 s and 72°C for 10 s; 60°C for 10 s, 70°C for 10 s. The relative gene expression was normalized with *Gapdh* and calculated using the 2^-ΔΔCt^ method [19]. The primer sequences used are displayed in Table 1.

**Table 1:** Primers used for RT-qPCR.

Genes	Primer	Sequence(5’-3’)
*Gapdh*	Forward primer	ACTCCACTCACGGCAAATTC
	Reverse primer	TCTCCATGGTGGTGAAGACA
*Fxr*	Forward primer	GGCAGAATCTGGATTTGGAATCG
	Reverse primer	GCCCAGGTTGGAATAGTAAGACG
*Acta2*	Forward primer	CACAGCCCTGGTGTGCGACAAT
	Reverse primer	TTGCTCTGGGCTTCATCCCCCA
*Col1a1*	Forward primer	TCCTGCGCCTAATGTCCACCGA
	Reverse primer	AAGCGACTGTTGCCTTCGCCTC
*Cyp7a1*	Forward primer	TCATCACAAACTCCCTGTC
	Reverse primer	TCACTTGGGTCTATGCTTC
*Bsep*	Forward primer	GAATGGACTGTCGGTATCTG
	Reverse primer	CAATGTTTGAACGGAGGAA
*Ntcp*	Forward primer	GGACAAGGTGCCCTACAAA
	Reverse primer	TGCCCACATTGATGACAGA
*Ostβ*	Forward primer	GAAGGAGCATCCTGGCAAAC
	Reverse primer	AGGAAGACCTGGCTGTTGTT

### Western blot

Total protein was extracted from frozen liver tissue (50 mg) using RIPA lysis buffer, protease inhibitor, and phosphatase inhibitor (P1082, Beyotime, Shanghai, China). The concentration of protein was determined with a BCA Protein Assay Kit (P0010, Beyotime, Shanghai, China). For western blot assay, the protein was resolved in SDSS gels and transferred to PVDF membrane (1620177, Bio-RAD, California, United States). After blocking with 5% milk for 3 h at room temperature, membranes were incubated with FXR primary antibody (25055-1-AP, Proteintech, Wuhan, China), α-SMA primary antibody (14395-1-AP, Proteintech, Wuhan, China), LC3 primary antibody (3868, Cell Signaling Technology, Boston, United States), Beclin-1 primary antibody (11306-1-AP, Proteintech, Chicago, United States), P62 primary antibody (18420-1-AP, Proteintech, Chicago, United States), and GAPDH primary antibody (60004-1-lg, Proteintech, Chicago, United States) at 4°C overnight. The anti-rabbit-IgG–HRP-conjugated antibody or anti-mouse IgG–HRP-linked antibody were used to incubate the corresponding membranes. The bands were visualized by adding chemiluminescent HRP substrate (BL520A, Biosharp, Beijing, China), captured by a ChemiDoc system (Bio-Rad, California, United States), and the gray intensity of each band was analyzed with Image J2x software. The gray intensity of FXR and α-SMA was used for correlation analysis. The relative expression levels of FXR and α-SMA were normalized to GAPDH, and the fold changes were calculated by comparing with the normal group. For LC3B, Beclin-1, and P62, they were normalized to GAPDH and then calculated versus the WT mice in the normal group.

### Statistical analysis

All data were expressed as Mean ± SEM. Statistical significance was analyzed with SPSS 19.0 software. Independent-sample *t*-test (two-tailed) was used to compare the two groups. One-way ANOVA with LSD or non-parametric test with Kruskal-Wallis H test was used for multiple groups. *P* <0.05 was considered statistically significant.

## Results

### The dynamic changes of FXR in livers of *S*. *japonicum* infected mice

**Fig 1:**
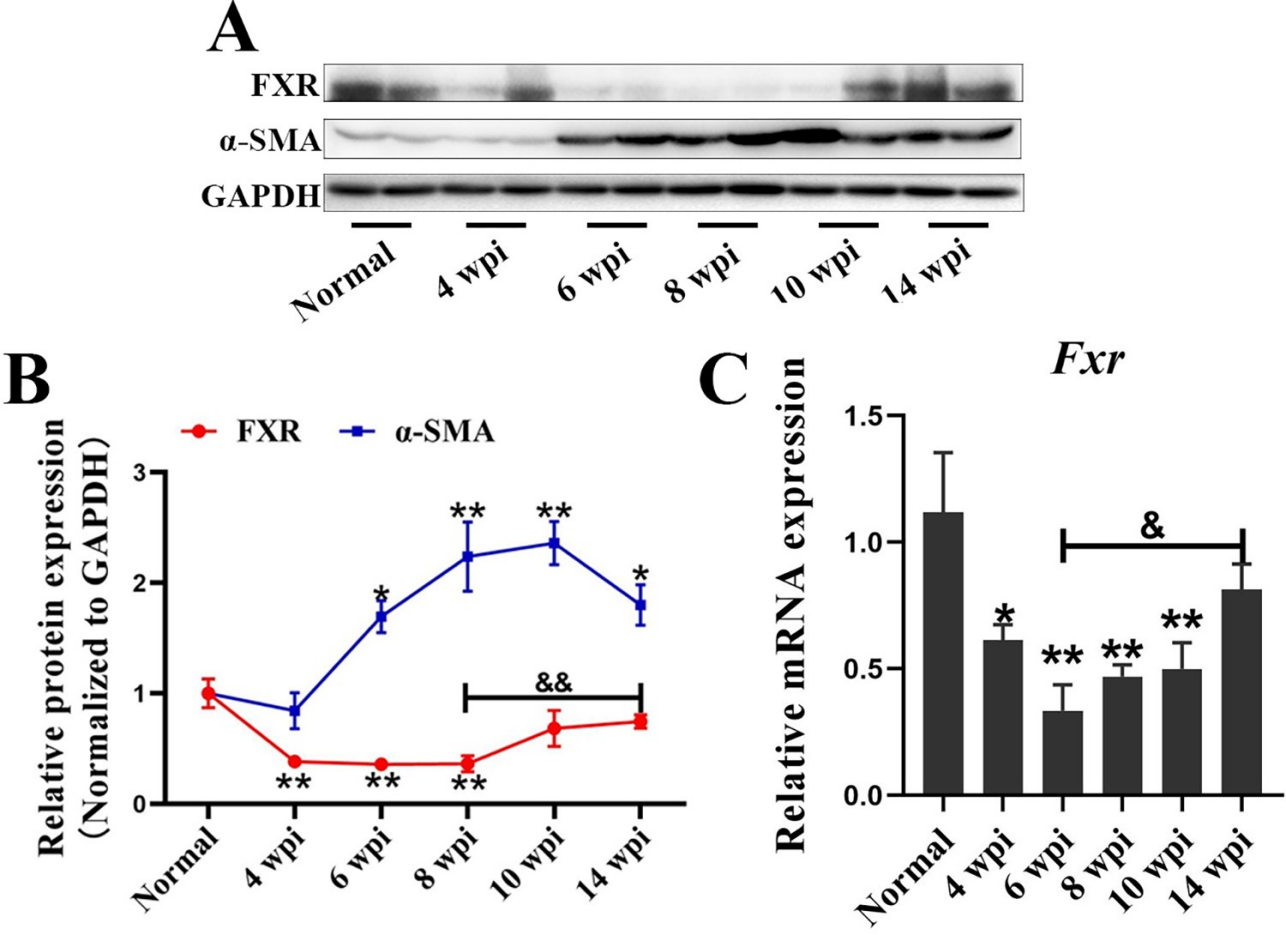
FXR decreases in mice livers with pathological injuries induced by *S*. *japonicum* infection. (A) Expression levels of hepatic FXR and α-SMA in mice infected with *S*. *japonicum* at different time-points were measured by western blot. (B) The relative expression levels of FXR and α-SMA were normalized to GAPDH, and the fold changes were calculated by comparing with the normal group. (C) The hepatic mRNA level of *Fxr* in mice infected with *S*. *japonicum* was detected by RT-qPCR. Data are presented as mean ± SEM. * *P* <0.05, infected groups *vs* normal group; ** *P* <0.01, infected groups *vs* normal group; ^&^*P* <0.05, ^&&^*P* <0.01.

The expression level of FXR was determined at 4 weeks, 6 weeks, 8 weeks, 10 weeks, and 14 weeks of *S*. *japonicum* infection. It was found that, compared with the normal group, the FXR protein level decreased significantly at 4 wpi (*P*<0.01), which lasted until 8 weeks after infection (*P*<0.01), and started to increase at 10 wpi. FXR was higher at 14 wpi than that in 8 wpi, and showed no difference with the normal group (*P*<0.01) (Fig 1A and 1B). In addition, the mRNA of *Fxr* was lower in 4 wpi (*P*<0.05), 6 wpi (*P*<0.01), 8 wpi (*P*<0.01) and 10 wpi (*P*<0.01) than in the normal group (Fig 1C). It also increased at 14 wpi and showed no difference with the normal group (Fig 1C). We have previously reported that *S*. *japonicum* infection resulted in pathological changes at 4 wpi. These pathological lesions remain to get worse until 10 wpi, and decreased at 14 wpi [20]. In Fig 1A and 1B, the expression of α-SMA, a marker of activated HSCs, was apparently increased at 6 wpi, 8 wpi, 10 wpi, and decreased at 14 wpi, which was consistent with what we have reported [20]. All these data indicated that the expression level of FXR was decreased in the livers of mice with pathological injuries caused by *S*. *japonicum* infection.

### FXR deficiency promotes the loss of body weight, death of mice, and liver injury caused by *S*. *japonicum* infection

**Fig 2:**
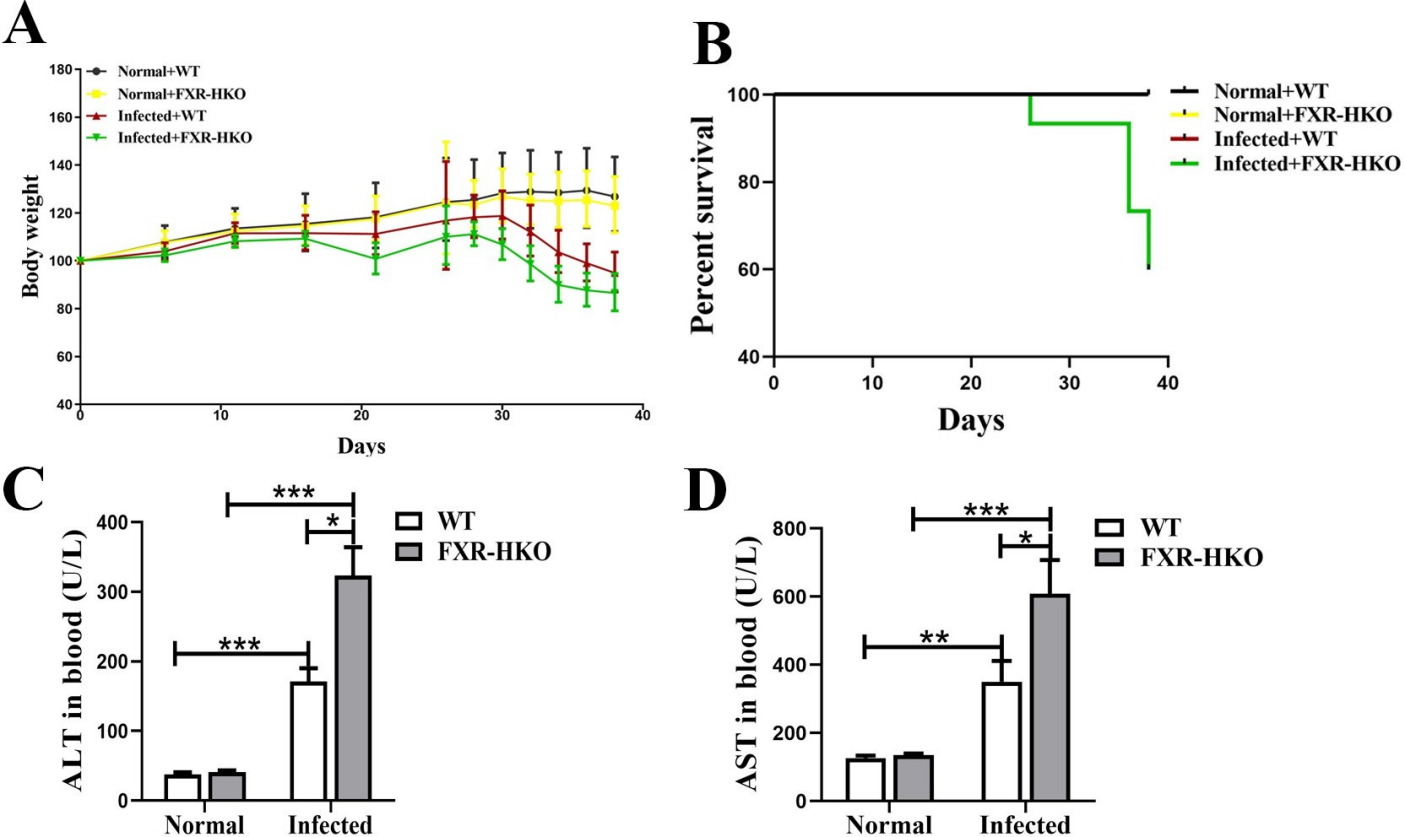
FXR deficiency promotes the loss of body weight, death of mice, and liver injury caused by *S*. *japonicum* infection. (A) The change ratio of body weight for mice in each group. (B) Survival rate of each group. (C) Serum analysis of ALT for mice in each group. (D) Serum analysis of AST for mice in each group. Data are presented as mean ± SEM. * *P* < 0.05, ** *P* < 0.01, *** *P* < 0.001.

To further investigate the role of FXR in hepatocytes in the progression of liver injury caused by *S*. *japonicum* infection, hepatocyte-specific FXR KO mice (FXR-HKO) were generated. It was found that the body weight of WT mice and FXR-HKO mice without infection all kept increasing. However, the body weight of WT mice began to decrease 30 days post-infection (Fig 2A). In comparison, the weight loss of FXR-HKO mice with infection was more obvious than that in the WT mice, and the onset time was also earlier in the FXR-HKO mice with infection (Fig 2A). Moreover, the death rate caused by an infection in the FXR-HKO group was higher than that in the littermate control group (Fig 2B). According to liver function observation, we also found that the ALT and AST were higher in FXR-HKO mice with infection than in littermate control mice (Fig 2C and 2D). Thus, these data suggested that FXR deficiency in hepatocytes promoted the progression of liver injury, and aggravated weight loss and death caused by infection.

### FXR deficiency has no effect on hepatic granulomas and fibrosis caused by *S*. *japonicum* infection

**Fig 3:**
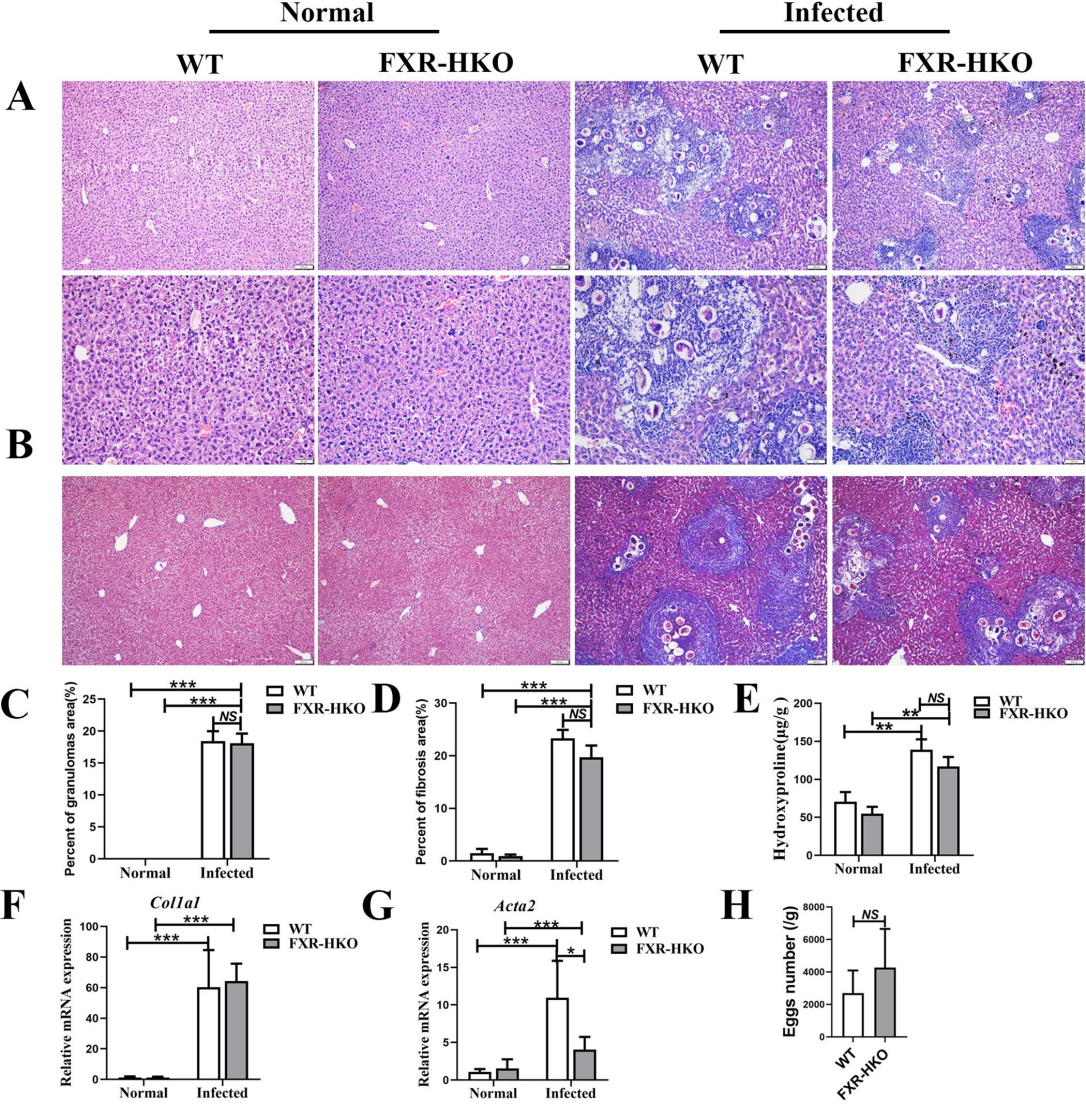
FXR deficiency does not affect hepatic granulomas and fibrosis caused by *S*. *japonicum* infection for 5 weeks post-infection. (A) Representative images of HE staining for each group. (B) Representative images of Masson staining for each group. (C) Percent of granulomas area was analyzed according to HE staining by Image Pro Plus 6.0 software. (D) Percent of fibrosis area was analyzed according to Masson staining by Image Pro Plus 6.0 software. (E) Liver hydroxyproline was detected by the alkaline lysis assay. The expression of *Col1a1* (F) and *Acta2* (for encoding protein α-SMA) (G) in liver were detected by RT-qPCR. (H) Eggs per gram in mice liver infected with *S*. *japonicum*. Data are presented as mean ± SEM. NS means no significance, * *P* <0.05, ** *P* <0.01, *** *P* <0.001.

Next, we investigated whether FXR deficiency in hepatocytes aggravated the outcome of schistosomiasis via modulating granulomas response and fibrosis. It was found that neither granulomas nor collagen deposition showed differences between WT and FXR-HKO mice (Fig 3A to 3D). FXR deficiency also didn’t influence the content of hydroxyproline and the mRNA level of *Col1a1* in livers of mice with infection (Fig 3E and 3F). However, after *S*. *japonicum* infection, it appeared that the expression of *Acta2*, which encoded the protein of α-SMA, was significantly decreased in the livers of infected FXR-HKO mice, compared with infected littermate control (WT) mice (Fig 3G, *P*<0.05). Besides, we found that FXR deficiency didn’t impact *S*. *japonicum* egg deposition in the liver (Fig 3H). Together, our data suggested that FXR deficiency in hepatocytes did not affect hepatic granulomas and fibrosis caused by *S*. *japonicum* infection at five week post-infection.

### FXR deficiency promotes inflammatory cytokines excretion caused by *S*. *japonicum* infection

**Fig 4:**
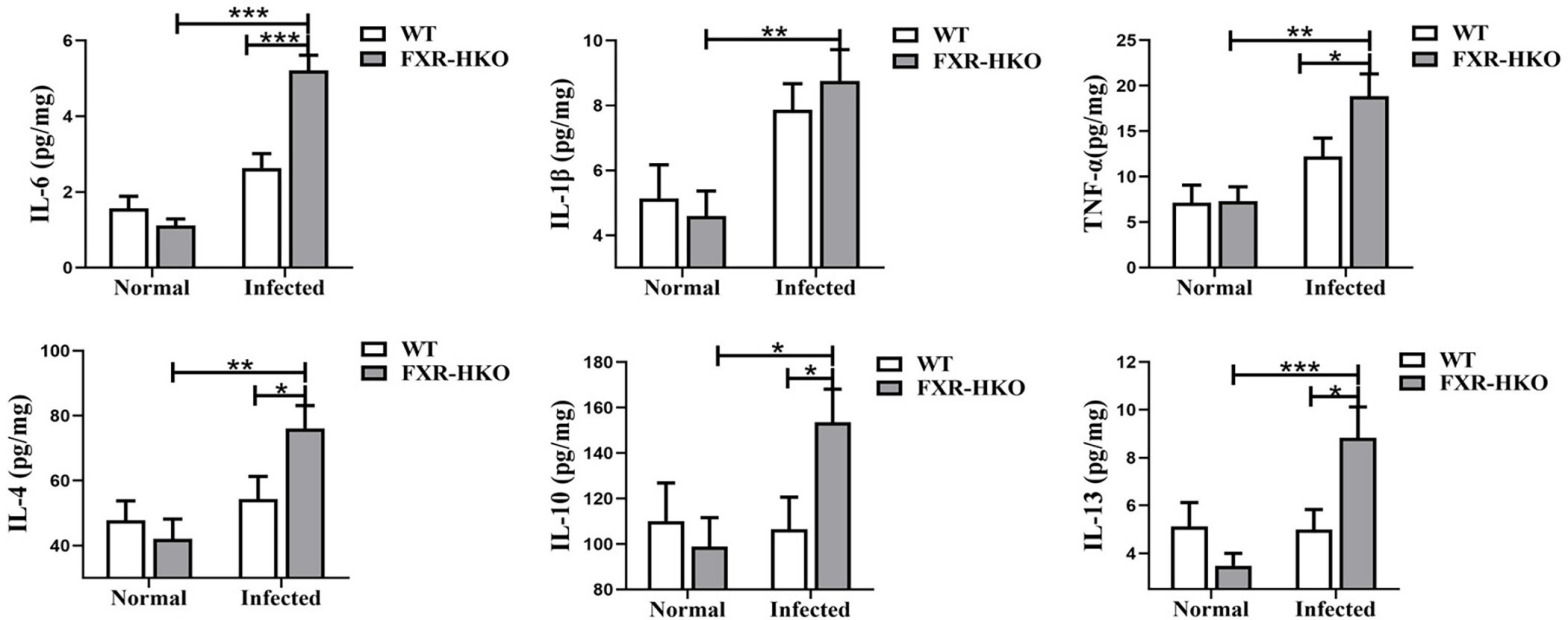
FXR deficiency promotes inflammatory cytokines production caused by *S*. *japonicum* infection. The levels of interleukin-6 (IL-6) (A), Interleukin-1β (IL-1β) (B), Tumor necrosis factor-α (TNF-α) (C), Interleukin-4 (IL-4) (D), Interleukin-10 (IL-10) (E), and Interleukin-13 (IL-13) (F) in liver from each group were detected by ELISA. Data are presented as mean ± SEM. *P <0.05, ** *P* <0.01, *** *P* <0.001.

FXR deficiency could promote the progression of schistosomiasis, which was supported by aggravated weight loss and death. Even though hepatic granulomas and fibrosis were not affected by FXR deficiency, we found that increased inflammatory cytokines were induced in infected mice with FXR deficiency. Briefly, in non-infected group, there were no obvious differences in IL-6, IL-1β, TNF-α, IL-4, IL-10, and IL-13 between WT mice and FXR-HKO mice. In comparison with WT infected group, FXR specific knock out in hepatocytes caused a further increase of IL-6 (*P*<0.001), TNF-α (*P*<0.05), IL-4 (*P*<0.05), IL-10 (*P*<0.05), and IL-13(*P*<0.05) in the liver of infected mice (Fig 4). The aforementioned data indicated that ALT and AST were increased in FXR deficiency mice with infection, which suggested more severe hepatocytes damage. Thus, it suggests that FXR specific knock out in hepatocytes aggravates the hepatocytes injury induced by *S*. *japonicum* infection, and triggers aberrant inflammatory responses in the liver.

### Bile acid homeostasis is disrupted in FXR-deficient mice with *S*. *japonicum* infection

**Fig 5:**
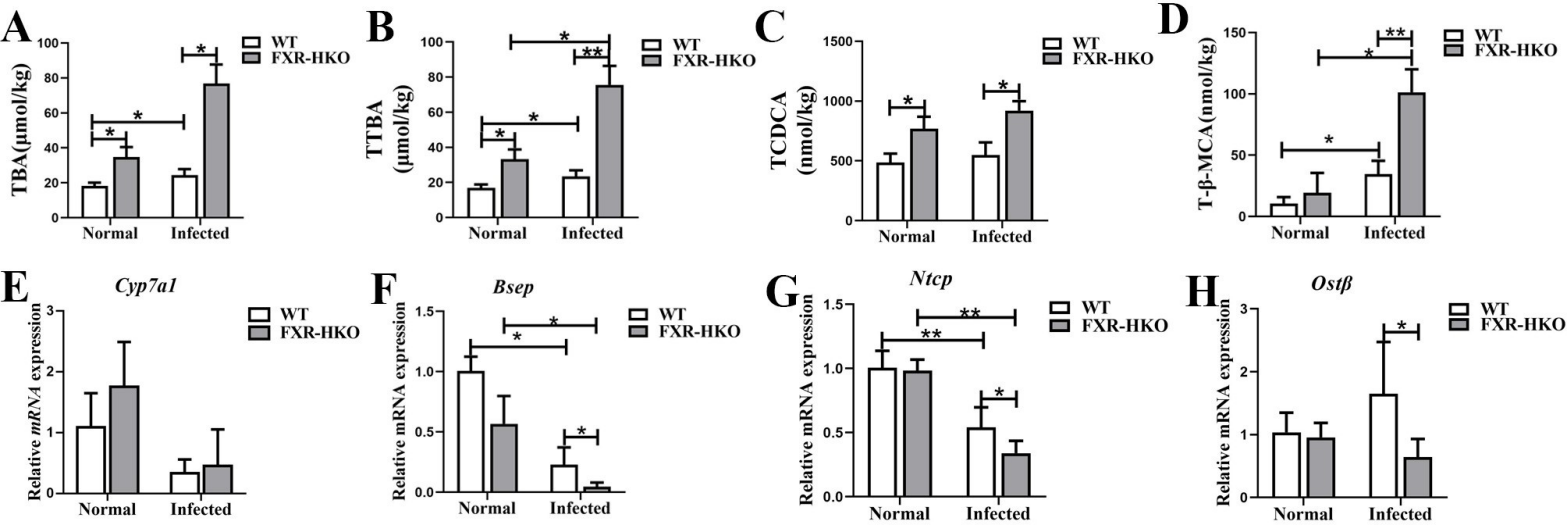
Bile acid homeostasis is disrupted in FXR-deficient mice with *S*. *japonicum* infection. The content of TBA (A), TTBA (B), TCDCA (C), and T-β-MCA (D) in mice liver from each group. The hepatic mRNA levels of *Cyp7a1* (E), *Bsep* (F), *Ntcp* (G), and *Ostβ* (H) in each group were detected by RT-qPCR. Data are expressed as mean ± SEM. * *P* <0.05, ** *P* <0.01.

To figure out whether FXR can potentially regulate the disrupted bile acid caused by *S*. *japonicum* infection or not, UPLC-MS/MS was used to determine the bile acid pool size. In WT mice, the total bile acid (TBA) in the liver was increased after *S*. *japonicum* infection. They were much higher in infected FXR-HKO mice, compared with WT mice with infection (Fig 5A, *P*<0.05). Moreover, the total taurocholated- conjugated bile acid (TTBA) (Fig 5B), taurochenodeoxycholic acid (TCDCA) (Fig 5C) and tauro-beta-muricholic acid (T-β-MCA) (Fig 5D) were higher in infected WT mice than normal WT mice, which was more pronounced in infected FXR-HKO mice (Fig 5C–5D). Interestingly, we found that the mRNA of *Cyp7a1*, a rate-limiting enzyme of bile acid synthesis, kept a similar level between WT and FXR-HKO mice (Fig 5E). However, *S*. *japonicum* infection decreased the expression of bile acids transporter-associated genes *Bsep*, *Ntcp*, and *Ostβ*, and FXR deficiency further repressed the expression of *Bsep*, *Ntcp*, and *Ostβ* (Fig 5F–5H, *P*<0.05). All these data indicated that FXR deficiency in hepatocytes elevated bile acids and aggravated hepatocytes injuries in mice of *S*. *japonicum* infection.

### Autophagy is inhibited in FXR-deficient mice with *S*. *japonicum* infection

**Fig 6:**
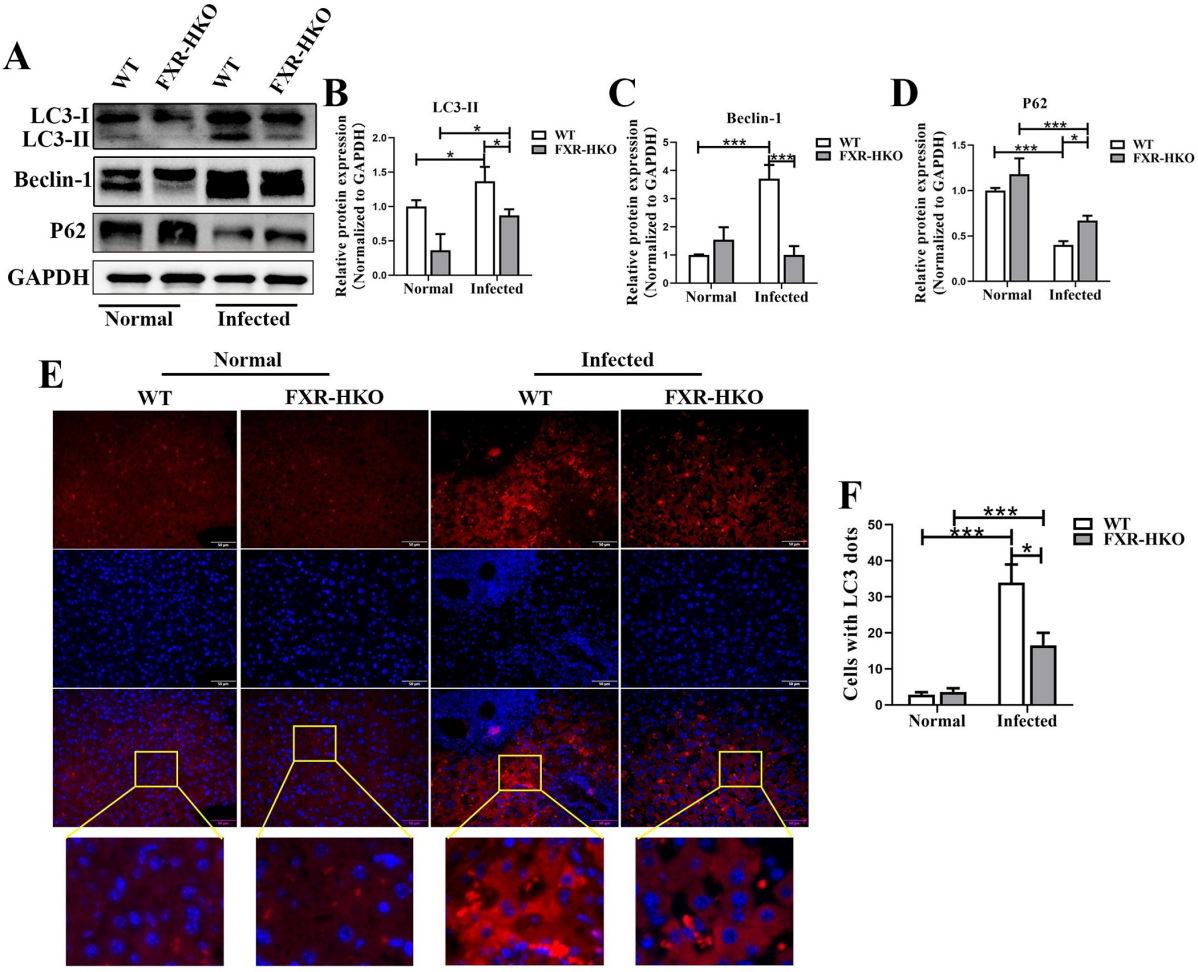
Autophagy is inhibited in FXR-deficient mice with *S*. *japonicum* infection. (A) Hepatic LC3, Beclin-1, and P62 in the mice of each group were measured by western blot. The densitometry analysis of LC3 (B), Beclin-1 (C), and P62 (D) was screened and measured by Image Pro Plus 6.0 software. (E) Immunofluorescene analysis of LC3 in liver tissue from each group, and the representative images were shown. (F) Counts of cells with LC3 dots were analyzed in mice for each group. Data are expressed as mean ± SEM. * *P* <0.05, *** *P* <0.001.

Increased bile acids have been reported to have cytotoxicity and could mediate effects on autophagy [21, 22]. We next explored whether hepatic autophagy was inhibited in FXR-deficient mice. It was found that LC3-II (*P*<0.05) and Beclin-1 (*P*<0.001) were higher in WT-infected mice than normal mice (Fig 6A–6C). But FXR deficiency in hepatocytes reversed the high expression of LC3-II and Beclin-1(Fig 6A–6C, *P*<0.05). P62, which is a substrate of autophagy and degrades during autophagy, significantly decreased in infected WT mice (Fig 6A and 6D, *P*<0.001), but it was comparatively higher in infected FXR-HKO mice (Fig 6A and 6D, *P*<0.05). According to immunofluorescence analysis, there was nearly no LC3 fluorescent signaling in hepatocytes in both WT and FXR-HKO normal mice (Fig 6E). But *S*. *japonicum* infection led to apparent LC3 signals in WT mice indicating enhanced autophagic flux (Fig 6E and 6F, *P*<0.001). FXR deficiency in hepatocytes could also decrease the level of the LC3 (Fig 6E and 6F, *P*<0.001). These data indicate that FXR deficiency impairs hepatic autophagy in *S*. *japonicum*-infected mice.

## Discussion

**Fig 7:**
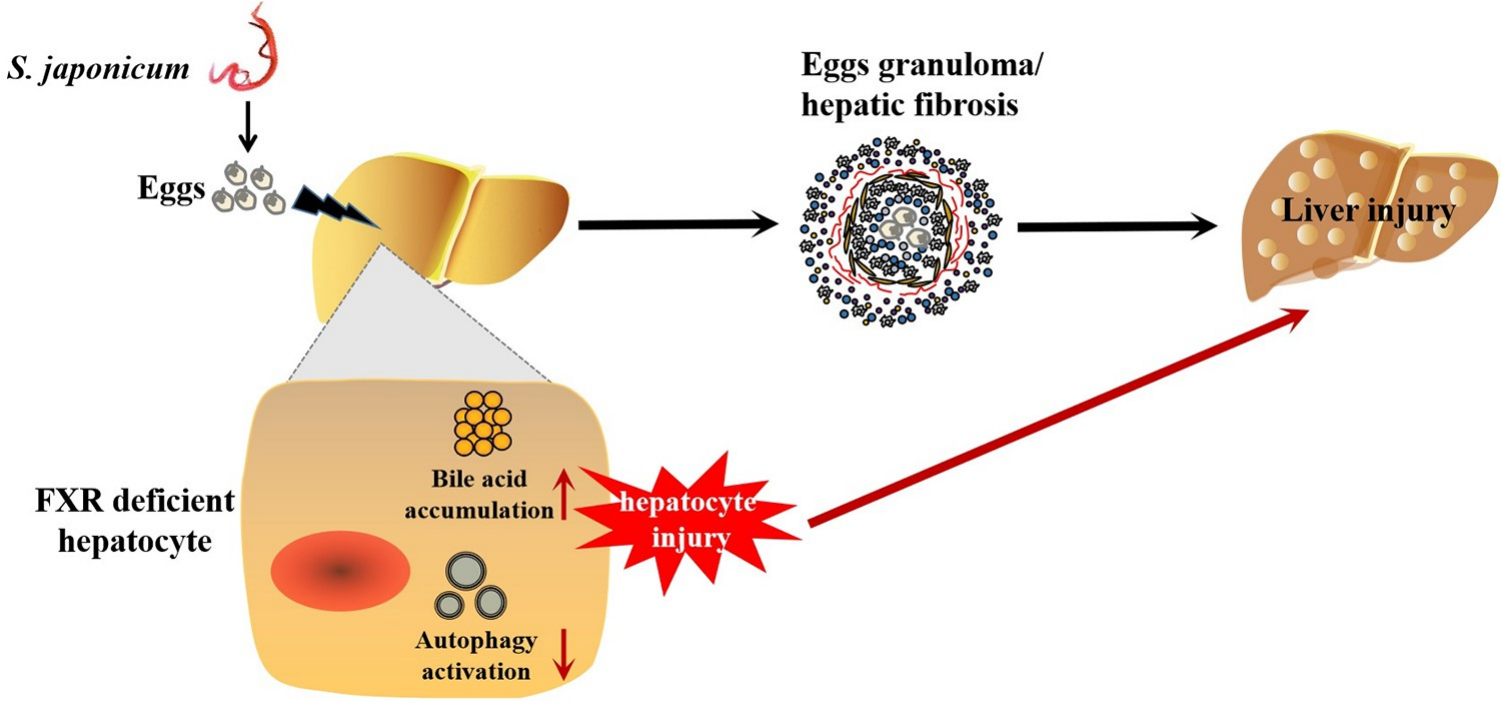
A schematic model shows that FXR deficient in hepatocytes increases bile acids toxicity and inhibits hepatocellular autophagy, which may therefore accelerate the progression of schistosomiasis by promoting hepatocytes injuries.

FXR, a master bile acid-activated receptor, functions as a key regulator in the uptake, transport, and excretion of bile acids in hepatocytes. Hepatic schistosomiasis results in inflammatory granulomas responses and fibrosis formation. However, little attention was paid to hepatocytes in the pathogenesis of schistosomiasis. In our present study, hepatocyte-specific FXR KO mice (FXR-HKO) were used to be infected with *S*. *japonicum* for 5 weeks. It was found that FXR deficiency did not affect hepatic granulomas and fibrosis caused by *S*. *japonicum* infection. However, it can promote hepatocytes damages via disrupting bile acid homeostasis and inhibiting autophagy probably, which eventually aggravates body weight loss, death, and liver injury caused by *S*. *japonicum* infection (Fig 7). The present study supports that bile acid/FXR in hepatocytes play a regulatory role in the progression of schistosomiasis.

We have previously reported that the hepatic pathology was the most severe during 6 weeks to 8 weeks post-infection, which could be self-relieved after 10 weeks [20]. In the present study, the expression profile of α-SMA was consistent with our earlier study above. It indicated that FXR decreased at the acute stage with serious liver pathological lesions, and increased with pathological remission. According to this data, we next investigated whether FXR could regulate the progression of schistosomiasis or not. FXR is dominantly expressed in parenchymal cells, and weakly in a small number of endothelial cells [23]. It is well-known that FXR in hepatocytes is the main regulator that modulates bile acid metabolism in cholestatic liver diseases [24]. However, the potential role of hepatocytes and the detailed mechanism have not been clarified in schistosomiasis. Based on this, hepatocyte-specific FXR KO mice were generated and infected with *S*. *japonicum* for 5 weeks. The data showed that infection with *S*. *japonicum* led to body weight loss and liver function injury. FXR deficiency in hepatocytes promoted the disease progression, which could be supported by earlier and more dramatic weight loss, and higher mortality in FXR-HKO mice than in WT mice.

The adult worms reside in the mesenteric veins and evoke inflammatory granulomas formation and secondary fibrosis by releasing eggs [25]. It was found that even FXR deficiency led to earlier onset time and more obvious weight loss, and higher mortality, but the hepatic granulomas and fibrosis were not affected at this stage of infection (5 wpi). It is well known that the granulomas and fibrosis occur perivascularly, its damage to hepatocytes is in an indirect manner, such as by blocking blood flow of nutrients to the hepatocytes [3]. So, the response and feedback of hepatocytes to granulomas and fibrosis may be also indirectly. Moreover, it was reported that eggs started to be deposited in liver at 4 wpi, and led to the formation of mature granulomas after 6 wpi in C57BL/6 mice, thus, the granulomas might be at an initial stage and becoming mature at 5 wpi [3]. Therefore, FXR in hepatocytes in such early stage may be not powerful enough to affect the progression of granulomas and fibrosis in hepatic schistosomiasis.

Bile acids are synthesized from cholesterol by cholesterol-7α-hydroxylase (CYP7A1) in hepatocytes, which are well known for their roles in promoting dietary lipid absorption. However, higher concentrations of bile acids are cytotoxic and pro-inflammatory in liver diseases like cholestatic and metabolic diseases [26]. Notably, FXR plays a key role in bile acid homoeostasis by controlling bile acids synthesis, transport, and excretion [26], which therefore reduce bile acids load and alleviate the pathogenesis of cholestasis [27]. Our data showed that bile acids overload in liver was induced by *S*. *japonicum* infection, which was aggravated by FXR deficiency in hepatocytes. However, bile acids synthesis associated rate-limiting enzyme CYP7A1 showed no changes between WT infected mice and FXR-HKO infected mice. Bile acids excreting into bile were through a bile salt export pump (BSEP), and into portal blood through organic solute transporter (OSTα/OSTβ). Conjugated bile acids are transported back from the intestine to the liver via sodium/taurocholate cotransporting polypeptide (NTCP). In our study, BSEP, NTCP, and OSTβ decreased more obviously in FXR-HKO infected mice than WT infected mice. All these data indicated that FXR deficiency resulted in bile acids overload in hepatic schistosomiasis via disturbing its transport but not synthesis [24]. Cholic acid (CA) and chenodeoxycholic acid (CDCA) are the two main primary bile acids. In mice, taurocholate- conjugated bile acids consist of more than 95% of total bile acids, which are more hydrophilic than the un-conjugated bile acids [7, 8]. In Fig 5B, it can be seen that the content of taurocholated-conjugated bile acid was almost similar with TBA. Apart from these, TCDCA and its secondary bile acid T-βMCA were more abundant in FXR-HKO infected mice than WT infected mice. T-βMCA is a novel antagonist of FXR [28], which can induce cell proliferation and DNA damage [29]. However, T-βMCA has little translational significance since all MCAs including T-βMCA are only present in the mice but absent in human. Nevertheless, our result at least indicated that FXR deficiency disturbed the bile acid metabolism in hepatic schistosomiasis.

Autophagy is a catabolic process that regulates the fundamental metabolism of cells and organs. As a housekeeping process, autophagy is the central point in regulating the homeostasis in different cell types in the liver [30, 31]. Here, we found that autophagy was significantly induced in hepatic schistosomiasis. However, FXR deficiency in hepatocytes impaired autophagy. It was reported that defective autophagy in liver parenchymal cells and non-parenchymal cells including Kupfer cells, and endothelial cells induce side effects in the liver disease such as non-alcoholic steatohepatitis [32]. However, impaired autophagy in hepatic stellate cells could attenuate liver fibrosis and hepatocellular carcinoma [32]. In drug-induced liver injury and nonalcoholic fatty liver diseases, hepatic autophagy is impaired; restoration of autophagy may be a potential strategy for treating these acute and chronic liver diseases [32]. It has been reported that overloaded bile acids block autophagy flux by inhibiting autophago-lysosomal maturation in an FXR-dependent manner [33]. In addition, hepatic deletion of autophagy-associated genes *Atg5* or *Atg7* lead to autophagy-deficient in mice, which promotes intracellular cholestasis with the increased level of bile acids [15]. All these works indicate a regulatory loop of FXR/bile acids and autophagy in the liver. In our present study, FXR depletion in hepatocytes manifested bile acid accumulation and impaired autophagy in *S*. *japonicum* infected mice liver [15]. These might be a “double whammy” for hepatocytes, which led to hepatocytes injury and therefore promoted the schistosomiasis progression. However, the detailed mechanism of how FXR/bile acids signaling modulated autophagy remains to be further studied.

In conclusion, the present study supports that FXR deficiency in hepatocytes increases bile acids toxicity and inhibits hepatocellular autophagy, which may therefore accelerate the progression of schistosomiasis by promoting hepatocyte injury. Our work provides a regulatory loop of FXR/bile acids-autophagy in schistosomiasis, which suggests a role of hepatic FXR in the protection from hepatic damages caused by infection with *S*. *japonicum*.
